# Differentiated Hypermedia E-Module for Social Studies (EduHYPE-IPS): Enhancing Creative Thinking Skills of Elementary School Students

**DOI:** 10.12688/f1000research.171479.1

**Published:** 2025-12-08

**Authors:** Yuyun Dwi Haryanti, Mohamad Syarif Sumantri, Mahpudin Mahpudin, Sapriya Sapriya, Hamdan Tri Atmaja, Sinta Maria Dewi, Okeu Santika, Lutvi Nurhadiansyah, Meisya Tania Putri

**Affiliations:** 1 Universitas Majalengka, Majalengka, West Java, Indonesia; 2 Universitas Negeri Jakarta, East Jakarta, Special Capital Region of Jakarta, Indonesia; 3 Universitas Pendidikan Indonesia Sekolah Pascasarjana, Bandung, West Java, Indonesia; 4 Universitas Negeri Semarang, Semarang, Central Java, Indonesia; 5 Universitas Buana Perjuangan Karawang, Karawang Regency, West Java, Indonesia; 6 Universitas Majalengka, Majalengka, West Java, Indonesia

**Keywords:** Differentiated learning, Hypermedia e-modules, Social Studies, Creative thinking, Elementary school

## Abstract

**Background:**

In the context of 21 ^st^ century education, creative thinking is a key competency required to navigate complex and rapidly changing global challenges. However, in the digital era, students’ creative thinking skills are often insufficiently developed due to the lack of attention to learning profiles, interests, and readiness. In social studies learning, this issue limits students’ opportunities to explore and express creative ideas.

**Methods:**

This study aimed to design and implement a hypermedia e-module based on differentiated learning, called EduHYPE-IPS, to enhance elementary school students’ creative thinking skills. The research employed a Research and Development (R&D) with ten stages the Borg and Gall model. The participants were fifth-grade students from six elementary schools in Majalengka and Yogyakarta, selected purposively. Data were collected through creative thinking tests and response questionnaires from students and teachers, and analyzed using descriptive and comparative statistics to determine validity, practicality, and effectiveness.

**Results:**

The developed e-module achieved a high level of validity, with an average expert validation score of 92.45% across media, content, and language aspects. Field trials demonstrated an improvement in students’ creative thinking, with an average N-Gain of 22.46% in the control class and 51.81% in the experimental class. Both students and teachers responded very positively, indicating that EduHYPE-IPS was engaging, user-friendly, and effective in supporting differentiated learning.

**Conclusions:**

The findings confirm that the hypermedia e-module meets the criteria of being valid, practical, and effective. EduHYPE-IPS can serve as an innovative digital learning resource to foster creativity in primary education and as a practical tool for teachers to design engaging, student-centered lessons integrating technology with differentiated teaching strategies.

## Introduction

The development of information and communication technology in the 21 ^st^ century has had a significant impact on the world of education ( Kaptan & Cakir, 2025; Rahimi & Oh, 2024; Rehman, 2025). Teachers are not the only source of knowledge, but rather act as facilitators who are able to utilize technology to create learning that is interactive, interesting, and relevant to students’ needs ( Dziubaniuk et al., 2023; Kormos, 2022; Mansour, 2024; Sitthiworachart et al., 2022). This study introduces an innovation in the form of a hypermedia-based e-module, integrating text, images, audio, video, and animation to enhance engagement and support students’ learning processes ( Bornman & von Solms, 1993; Benfarha et al., 2025; Gril et al., 2025; Ikram et al., 2024; Jobim & Giraffa, 2024; Lim & Karatza, 2024).

Social Science learning in elementary schools requires interactive media to help students grasp abstract concepts and connect them with real-life contexts ( Hasni et al., 2025; Susanto et al., 2022; Syawaluddin et al., 2020). However, in reality, social studies learning in many schools still tends to be conventional, teacher-centered, and minimal use of digital technology ( Fitchett & Heafner, 2018; Golightly & Sprenger, 2024; Mengiste, 2025; Yilmaz, 2008). Such traditional approaches often focus on rote memorization rather than inquiry and problem-solving, which limits opportunities for students to think critically, express creativity, and collaborate ( Burgos-Videla et al., 2025; Sharma, 2025). As a result, students are not only less engaged and easily bored, but they also face long-term consequences, including lower learning achievement, underdeveloped creative and problem-solving skills, and inadequate preparation for global competencies required in the 21st century ( Daniel et al., 2024; Wadtan et al., 2024). This highlights the urgency of developing innovative learning media that can transform social studies learning into a more meaningful and future-oriented experience.

In the digital era, the ability to think creatively is one of the essential competencies that need to be trained from an early age ( Dilekçi & Karatay, 2023; Nikkola et al., 2024; Segundo-Marcos et al., 2025). However, differences in students’ learning readiness, interests, and learning profiles are often not accommodated in the learning process ( Chou-Lee & Tran, 2025; Gheyssens et al., 2022; Goyibova et al., 2025; Zhong, 2023). This results in learning being less relevant to individual needs, low motivation to learn, and limited exploration of creative ideas. Differentiated learning is a solution to accommodate student diversity by adapting content, processes, and learning products according to their individual characteristics ( Tomlison, 2021; Tomlinson, 2017; Tomlinson et al., 2003).

The application of hypermedia e-modules built on differentiated learning is an urgent solution because it can present material that is interactive, adaptive, and according to the learning characteristics of students. These e-modules allow teachers to provide a rich learning experience through the integration of text, images, audio, video, and animation, while tailoring learning to individual students’ differences. Thus, the development of this media has a high urgency to answer the challenge of social studies learning that is more effective, relevant, and oriented towards strengthening creative thinking skills in elementary schools.

Various previous studies have developed digital and hypermedia-based e-modules for student learning. Most of the research focused on improving cognitive learning outcomes ( Alyusfitri et al., 2024; Logan et al., 2021; Khotimah et al., 2021; Wagino et al., 2024), Critical Thinking ( Pertiwi et al., 2024), motivation ( Delita et al., 2022), and Understanding concepts ( Adawiyah et al., 2020; Lestari et al., 2022). However, not many have specifically integrated the principle of differentiated learning into the hypermedia e-module. In fact, differentiated learning has great potential to accommodate the diversity of students’ learning readiness, interests, and learning profiles ( Gheyssens et al., 2022; Goyibova et al., 2025).

Research that integrates hypermedia e-modules with differentiated learning approaches in elementary social studies is still very limited, particularly in exploring their impact on students’ creative thinking skills ( Kioko et al., 2025; Rochmattulloh et al., 2022). Most previous studies have concentrated primarily on conceptual mastery, overlooking creative thinking, which is a core competency of the 21st century ( Ajlouni, 2021; Daeli et al., 2025). This gap is critical, because without fostering creativity, students may achieve short-term academic goals but remain unprepared for broader demands beyond the classroom. Creative thinking is not only a cognitive outcome but also a crucial ability that equips students to adapt to rapid technological change, compete in a global economy, and contribute to solving complex social problems. Therefore, this research aims to close the gap by creating and evaluating a differentiated hypermedia-based e-module in social studies learning, with the specific aim of enhancing students’ creative thinking skills as a foundation for lifelong learning and global competence.

The product developed in this study is a differentiated hypermedia e-module specifically designed for elementary social studies learning. Unlike conventional digital modules that generally present static content, this hypermedia e-module integrates multiple media formats text, images, audio, video, animation, and interactive hyperlinks allowing students to navigate learning materials non-linearly according to their needs and interests. What makes it distinctive is the incorporation of differentiated learning principles, where the content and activities are designed with multiple pathways and varying levels of complexity to take into account the various learning styles, readiness levels of students, and interests. In this way, the e-module not only delivers information but also provides interactive tasks and problem-based activities that stimulate students’ creative thinking. The validity of the module is examined through expert judgment, its practicality through teacher and student responses, and its effectiveness through improvements in students’ creative thinking skills. Thus, this study not only addresses a critical gap in previous research but also contributes a concrete innovation in the form of a differentiated hypermedia e-module for social studies instruction in elementary schools.

The development of differentiated hypermedia e-modules is expected to be one of the effective solutions in social studies learning. This media not only presents material in an interactive and interesting manner, but also provides chances for pupils to study in accordance with their learning style, interests, and ability level. With these characteristics, this research has urgency to be carried out, because it is expected to be able to produce differentiated based social studies hypermedia e-modules that not only enhance elementary school pupils’ capacity for creative thought while also helping to raise the standard of instruction as a whole.

## Methods

### Research design

**Figure 1.**
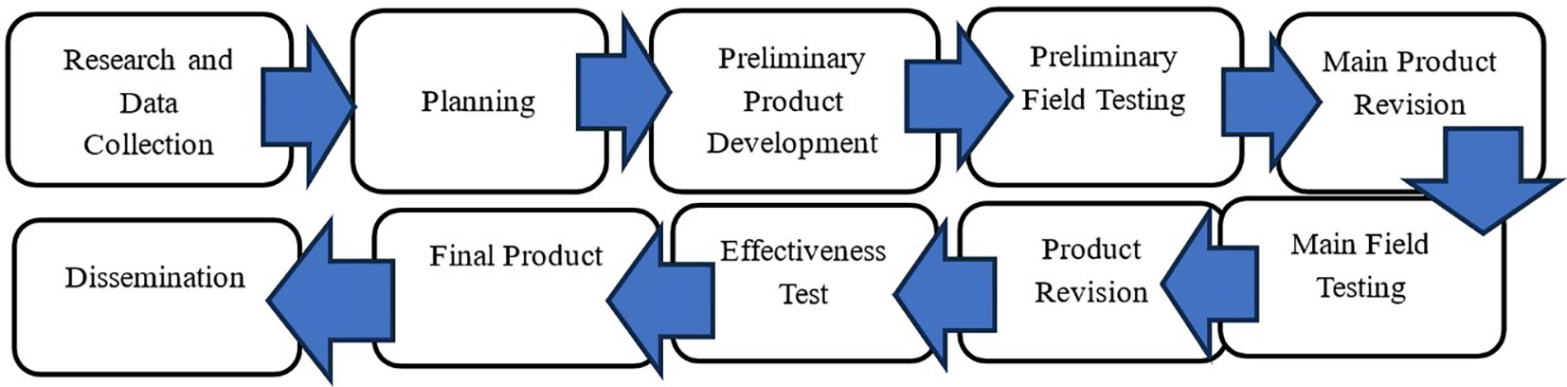
Borg & Gall research steps.

The research design used in this study is research and development (R&D) which is developed using the Borg and Gall model by selecting only ten stages, namely: (1) research and data collection, (2) planning, (3) preliminary product development, (4) preliminary field testing, (5) main product revision, (6) main field testing, (7) product revision, (8) effectiveness test, and (9) final product, and (10) dissemination ( Gall et al., 2003). This adapted was necessary to adapt to the context of developing a hypermedia e-module in elementary social studies, ensuring that the research process remains systematic while being realistic to the limitations of time and resources, and still guaranteeing the validity, practicality, and effectiveness of the product. This model was chosen because it is suitable to produce products in the form of differentiated learning-based hypermedia e-modules that are valid, practical, and effective for use in social studies learning in elementary schools. The steps of the research carried out with reference to Borg & Gall can be seen in the following Figure 1.

Figure 1 shows that the *first step* of research and data collection includes empirical studies related to differentiated social studies learning conditions in elementary schools through literature studies, analysis of teacher and student needs, and studies of elementary school social studies curriculum to determine learning problems and the need for hypermedia e-modules. *Second*, planning, namely the preparation of drafts by determining learning outcomes, materials, and design of Hypermedia e-modules in differentiated social studies learning in elementary schools. *Third*, initial product development, namely constructing prototypes that are validated by material experts, linguists, media experts (academics and practitioners) to determine the feasibility of the product who were selected based on their academic qualifications, teaching experience of more than five years, and relevant expertise in instructional design social studies and educational technology. These criteria ensured that the validators possessed sufficient competence and authority to provide a credible assessment of the product.

*Fourth*, the initial product trial is to conduct limited tests in two 5th grades, namely SDN SDPL and SDN KRO Majalengka district, to maintain confidentiality and adhere to research ethics, the full names of the participating schools are not disclosed in this manuscript. Instead, abbreviations (e.g., SDN SDPL, SDN KRO) are used consistently to protect the identity of the institutions without reducing the clarity of the findings. *Fifth*, the initial product revision is to revise draft 1 through a limited test to draft 2. *Sixth*, product trials, namely conducting large-scale tests of draft 2 results in two 5th grades, namely SDN SLW and SDN CMS Majalengka district. These schools represent public elementary schools in semi-urban areas with adequate but limited digital resources, where differentiated learning relies mainly on basic ICT facilities such as projectors and computer.

*Seventh*, product revision, namely revising draft 2 to draft 3. *Eighth*, the effectiveness test of draft 3 was carried out by implementing differentiated hypermedia e-modules in social studies learning, measuring the improvement of students’ creative thinking skills and the practicality of teachers and students at SDN PJ and SDN TR in Gunung Kidul district, Yogyakarta. These schools are located in rural areas with diverse socio-economic backgrounds, modest digital readiness, and a curriculum environment aligned with the national policy that encourages the integration of ICT and innovative learning media. *Ninth*, the final product, which is the final revision of the draft product 4. *Tenth*, publication of articles.

### Participants

Research participants used with *purposive sampling techniques* mean paying attention to certain school criteria, namely schools that implement the independent curriculum. Six experts served as validators: two in social studies content, two in media, and two in language ( Nabhan & Habók, 2025). The number of experts was considered sufficient as it represents a balanced triangulation of perspectives across the three domains most relevant to the hypermedia e-module (content, media, and language). The experts were selected based on their academic qualifications (at least a master’s degree in education or relevant fields), more than five years of teaching or professional experience, and proven expertise in instructional design, educational technology, or language studies. These criteria ensured that the validators had both competence and authority to provide credible judgments. The users involved were 19 grade 5 elementary school teachers, and the field trial respondents were 184 grade 5 students from Majalengka district, West Java, and Gunung Kidul district, Yogyakarta. The students were between 10 and 11 years old, with a relatively balanced gender distribution (male and female), and came from mixed socio-economic backgrounds that reflect both semi-urban (Majalengka) and rural (Gunung Kidul) contexts. This demographic diversity provided a representative sample for testing the practicality and effectiveness of the developed hypermedia e-modules.

### Data collection

The data collection technique was carried out by: 1) questionnaires for expert validation, teacher and student practicality; 2) The creative thinking ability test is used to measure the effectiveness of the product covering four aspects, namely: (a) Fluency, (b) Flexibility, (c) Originality, and (d) Elaboration ( Anam et al., 2023; Nufus et al., 2024); and 3) observations, documentation studies and interviews as supporting data. The data analysis technique was carried out through validity analysis of validator scores with the Likert scale, practicality analysis based on the results of teacher and student questionnaires, and effectiveness analysis based on statistical tests t-test and N-gain to determine the improvement of students’ creative thinking skills based on Hake calculations ( Hake, 1999).

### Instruments

The validity analysis was carried out using validator scores and user questionnaire responses assessed with a 4-point Likert scale, where 1 (not good/not practical), 2 (less good/less practical), 3 (good/practical), and 4 (very good/very practical) ( Crawford et al., 2025). The scores from the validators were then averaged to determine the validity category of the product. The scores from the validators were then averaged and converted into percentages using the formula (total score obtained ÷ maximum possible score) × 100%. The percentage results were used to determine the validity category of the product, where higher percentages indicated higher levels of validity. The resulting percentages were then interpreted based on four categories: 0–25% (Not valid/Not practical), 26–50% (Less valid/Less practical), 51–75% (Valid/Practical), and 76–100% = (Very valid/Very practical) ( Crawford et al., 2025).

The practicality of EduHYPE-IPS was measured through teacher and student response questionnaires. For students, the aspects assessed included attractiveness, ease of understanding the material, alignment with differentiated learning, enhancement of creative thinking, and learning motivation. For teachers, the aspects covered alignment with differentiated learning, design and features, support for creativity development, and ease of use. Thus, practicality was viewed in terms of simplicity, attractiveness, and effectiveness in supporting interactive and innovative social studies learning.

### Data analysis

**Figure 2.**
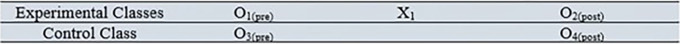
Trial using *pretest-posttest control group design.*

The effectiveness of the EduHYPE-IPS was determined by comparing pretest and posttest scores using the n-gain criterion, with values in the range of 0.3 ≤ (g) ≥ 0.7 indicating moderate to high effectiveness. In this study, the assignment of experimental and control groups was based on existing class structures due to institutional constraints. This limitation is acknowledged as a weakness of the study, as it may affect the generalizability of the findings. Nevertheless, the results consistently demonstrate that the use of the differentiated Hypermedia E-Module is effective in improving students’ creative thinking skills. Testing of Hypermedia E-Module products using *the experimental design of pretest-posttest control group* ( Creswell & Creswell, 2018) is shown in Figure 2.

Information:

**Table:** 

O _1_	Pretest score *of* the experimental class on students’ creative thinking skills before using the Hypermedia E-Module in differentiated social studies learning (EduHYPE-IPS).
O _2_	Posttest scores *of* experimental classes on students’ creative thinking skills after using the Hypermedia E-Module in differentiated social studies learning (EduHYPE-IPS).
O _3_	Pretest score *of* the class control of students’ creative thinking ability before using the E-Module in social studies learning.
O _4_	Posttest score *of* the class control of students’ creative thinking ability before using the E-Module in social studies learning.

### Ethical consideration

This study was conducted in accordance with ethical research standards. Approval for the research procedures, including data collection involving elementary school students, was obtained from the Research and Community Service Institute (LP2MI) of Universitas Majalengka with the certificate number: K.194/LP2MI UNMA/V/2025 on May 25th, 2025. Written informed consent was obtained from the school principals, teachers, and parents of participating students. All participants were assured of the confidentiality and anonymity of their data, and participation was voluntary. No coercion or external pressure was applied in obtaining consent. In addition, to protect participants’ rights and privacy, all personal data collected during the research were anonymized, and all records and digital data were stored securely. Only the research team had access to the data, which will remain confidential and used solely for academic purposes.

### Informed consent statement

Prior to data collection, the researcher obtained formal approval for conducting the study through a Research Partner Consent Letter signed by the participating institution, indicating institutional consent and support for the research activities. All participants were fully informed about the objectives, significance, and procedures of the study before participation. Participation was entirely voluntary, and participants retained the right to withdraw at any time without any consequences. The confidentiality and anonymity of all participants were strictly maintained, and all data collected were used exclusively for academic and research purposes.

## Results and discussion

### Results

**Research and data collection**, it was found that the results of literature study analysis and needs analysis through questionnaires and interviews with grade 5 teachers on social studies learning in elementary schools, information was obtained that social studies learning is still dominant using lecture and textbook methods. Teachers complain that existing interactive media, such as slides, videos, or basic learning apps, are insufficient to address diverse learning styles. Although these tools have been tried, they are often ineffective as students quickly lose interest and the materials fail to support differentiated learning. This condition shows the need for the development of differentiation-based hypermedia e-modules that are interactive and according to the needs of students.

**Figure 3.**
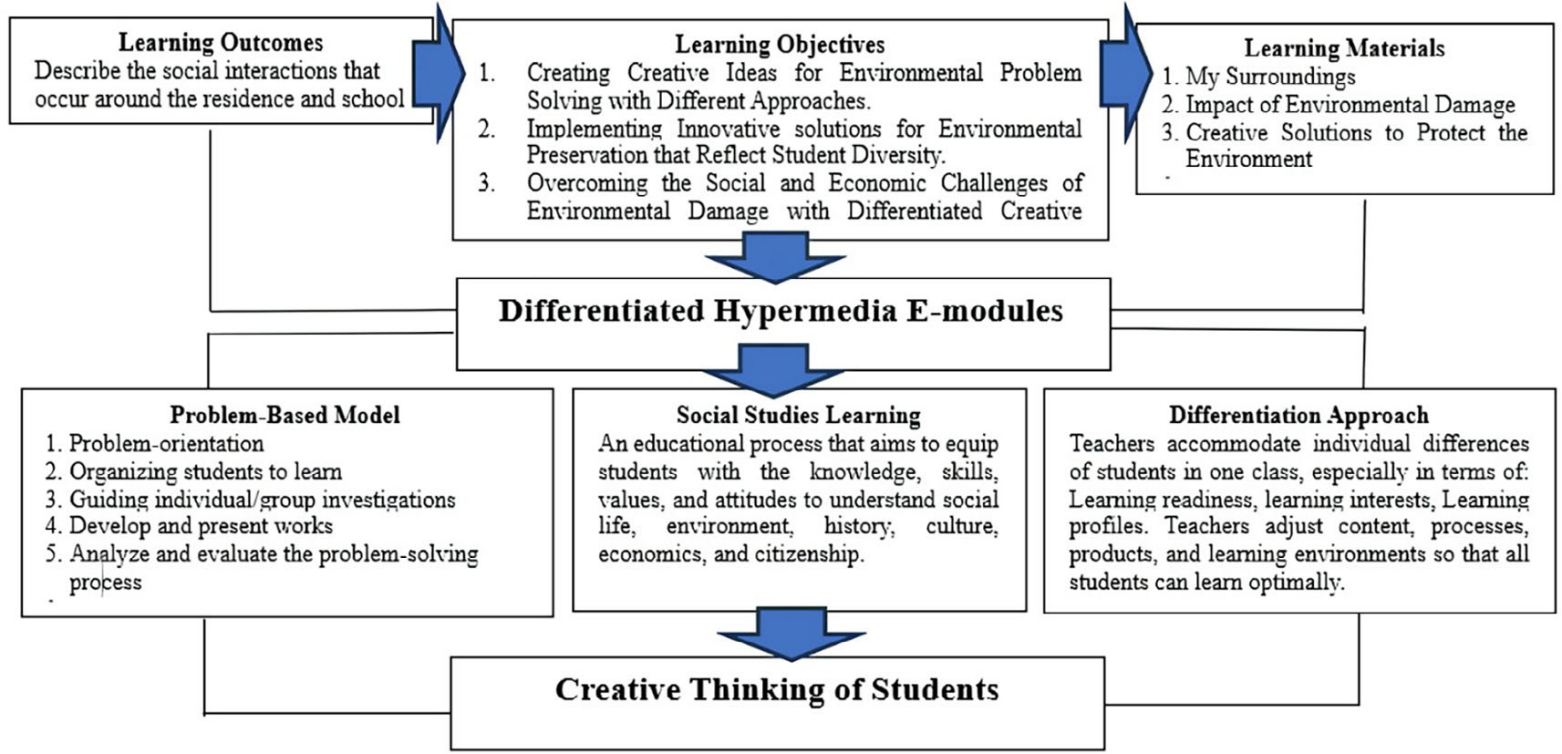
Stages of differentiation-based hypermedia e-module planning.

**Planning**, at this stage, the initial design of the product is prepared, including: learning outcomes (CP), learning objectives, learning materials, and e-module design which is equipped with videos, animations, images, interactive quizzes, and flexible navigation features. Differentiated learning strategies are applied by providing a selection of materials, exercises, and activities according to students’ interests, learning styles, and ability levels. The stages of differentiation-based hypermedia e-module planning in social studies learning can be seen in Figure 3.

**Figure 4.**
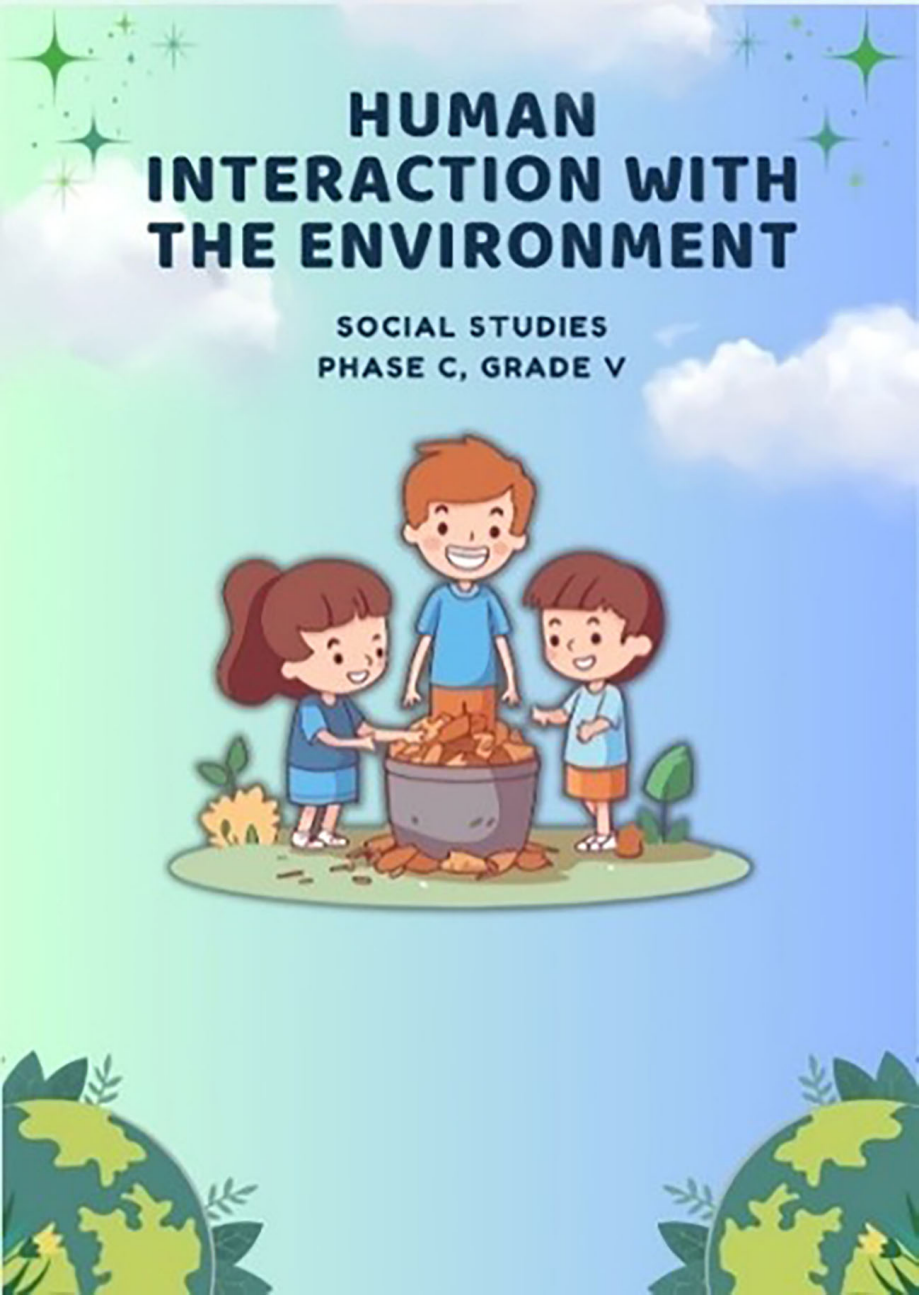
Prototype e-modul hypermedia.

**Initial Product Development**, Prototype hypermedia e-modules are developed using supporting software. The initial product can be seen in the following Figure 4.

Figure 4 above, shows that the 5th grade social studies material with the theme “Human interaction with the environment” uses interactive features using the application Canva, youtube, qr code, sketchfab, educaplay, elevenlabs, social media (facebook, instagram, tiktok), google form, and wordwall. Interactive features in the form of short videos, game-based materials and quizzes with content differentiation (varied materials), processes (activities based on interests), and products (learning outcomes differ).

**Initial Product** Trials, Initial products were validated by six academic experts and practitioners, namely material experts, media experts, and linguists. The results of expert validation related to differentiated hypermedia e-modules in social studies learning (EduHYPE-IPS) can be seen in Table 1.

**Table 1.** Recapitulation of expert validation analysis results.

Validation	Expert	Score (%)
1	Material (SPY)	96,88
2	Material (HTA)	90,63
3	Media (PY)	92,19
4	Media (AR)	95,31
5	Language (AP)	90,63
6	Language (YA)	89,06
Average		92,45

**Figure 5.**
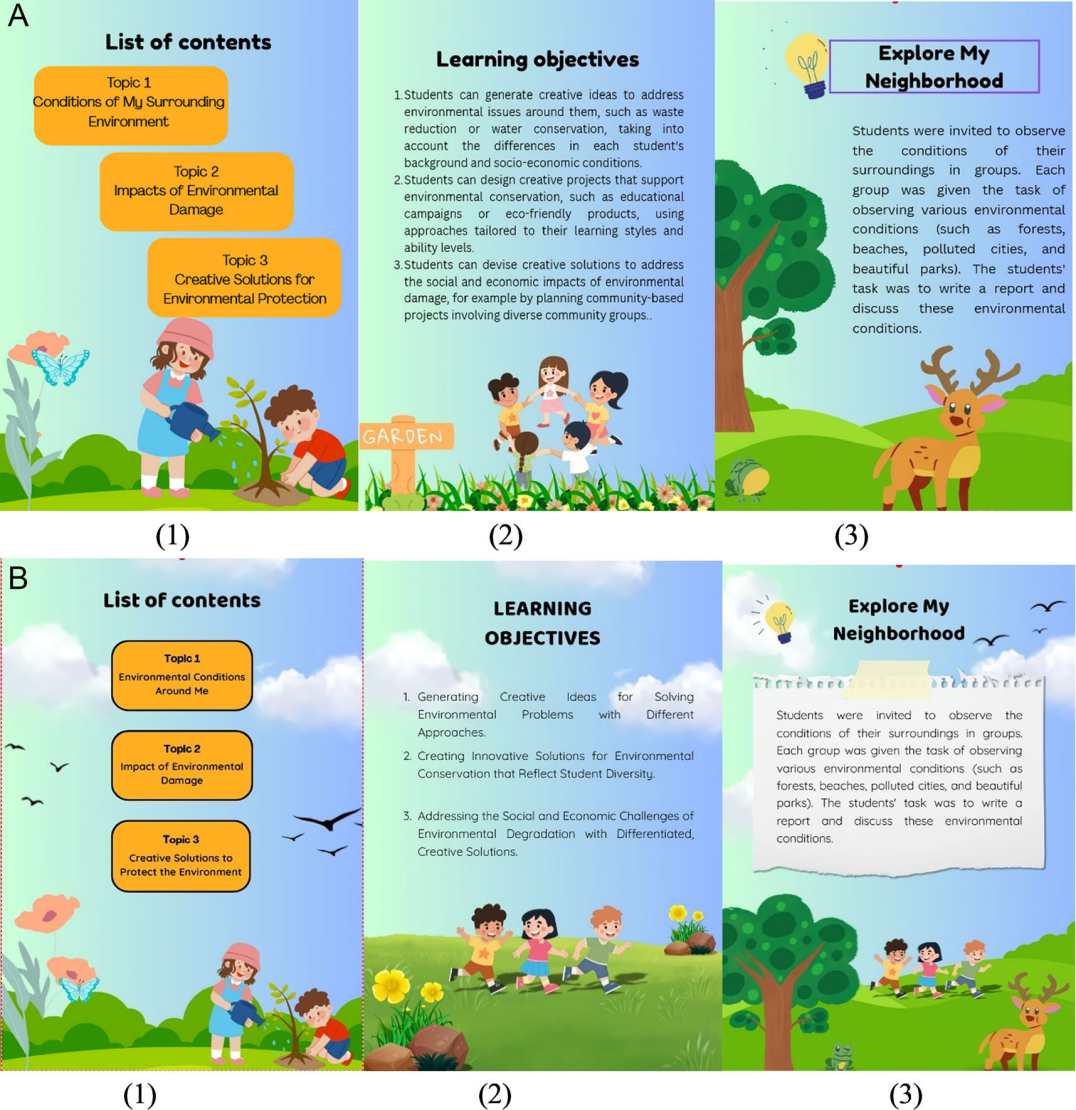
(A) Initial product results. (B) Initial product revision results.

The validation results in Table 1, the results show that the mean score is 92.45%, this is included in the category of very valid. The validity interpretation is based on the following criteria: 0–25% = not valid, 26–50% = less valid, 51–75% = valid, and 76–100% = very valid, meaning that the developed product is very suitable for use, but there are slight revisions to improve the hypermedia e-module product ( Haryanti et al., 2025a). Expert input includes: 1) learning objectives; 2) simplification of navigation display; 3) grammar improvements. Initial Product Revision, based on validator input, improvements were made to train students’ creative thinking. The results of the comparison of the initial product and the improvement results can be seen in the following Figure 5 (A) Initial Product Results Figure 5(B) Initial Product Revision Results.

Figure 5A shows the initial product results and then revised according to the validator’s input. Figure 5B shows the results of correcting input from validators to revise learning objectives, simplify navigation displays, and improve grammar. After revision, the product is declared worthy of trial.

**Product Trials**, Limited Trials were carried out in two schools, namely SDPL and KRO involving 2 5th grade teachers and 60 students. The limited-scale product trial’s outcomes are displayed in Table 2.

**Table 2.** Limited scale product trial results.

Criteria for creative thinking	SDPL	KRO
Very high	23	19
High	6	9
Low	1	2
Not creative	0	0

The Table 2 shows the level of student creativity in two schools, namely Public elementary school (SDPL) and Public elementary school (KRO) which are grouped into four categories, namely very high, high, low, and not creative. The results of observations showed that most of the students from the two schools were in the very high creative category, with a total of 23 students in Public elementary school (SDPL) and 19 students in Public elementary school (KRO). Furthermore, in the high creative category, there are 6 students from Public elementary school (SDPL) and 9 students from Public elementary school (KRO). Meanwhile, in the low creative category, there were only 1 student from Public elementary school (SDPL) and 2 students from Public elementary school (KRO). Interestingly, no students were included in the category of not creative in either Public elementary school (SDPL) or Public elementary school (KRO). Thus, it can be concluded that the majority of students in both schools demonstrated very high creative thinking skills, some were in the high category, and only a few were in the low category, with no students classified as uncreative. The categorization of creative thinking skills was determined based on score ranges, namely Very high (81–100), high (61–80), moderate (41–60), and low (21–40), and very low (0–20).

**The practicality test** was carried out to learn how instructors and students responded. The assessment aspects of the Likert scale 1-4 teacher response questionnaire include: 1) the suitability of the module with differentiated learning; 2) the design and features of EduHYPE-IPS; 3) the development of creative thinking; and 4) feasibility and ease of use. The results of the analysis of teachers’ responses regarding the practicality of using EduHYPE-IPS can be seen in Table 3.

**Table 3.** Teacher response questionnaire.

Teacher	Rate	Percentage (%)	Teacher	Rate	Percentage (%)	Teacher	Rate	Percentage (%)	Teacher	Rate	Percentage (%)
1	3,8	95	6	3,6	91	11	3,9	97	16	3,5	88
2	4,0	100	7	3,8	94	12	3,9	97	17	3,6	91
3	3,9	98	8	3,6	89	13	3,9	97	18	3,7	92
4	3,7	92	9	3,4	86	14	3,7	92	19	3,7	92
5	3,4	86	10	4,0	100	15	3,6	89	**Rate**	**3,7**	**92,9%**

Based on the results Table 3 of the teacher’s response questionnaire, a score was obtained with a range of 3.4 to 4.0. In general, most scores are in the range of 3.6 to 3.9 with an average percentage of teacher responses reaching 92.9%, so it can be categorized as very good. This percentage was obtained by converting the average score on a 4-point Likert scale into a percentage using the formula: (total score obtained ÷ maximum possible score) × 100%. This shows that the EduHYPE-IPS developed is considered very practical to use in social studies learning. This shows that the EduHYPE-IPS implemented is in accordance with the teacher’s expectations. Thus, it can be concluded that EduHYPE-IPS has a high level of practicality, making it feasible to use in social studies learning in elementary schools.

The assessment aspects of the likert scale 1-4 student response questionnaire include: 1) the attractiveness of EduHYPE-IPS; 2) the ease of understanding the material; 3) differentiated learning; 4) increased creative thinking; 5) comfort and motivation to learn. The results of the analysis of student responses related to the practicality of using EduHYPE-IPS can be seen in Table 4.

**Table 4.** Student response questionnaire.

Student	Rate	Percentage (%)
1-30	3,87	96,65
31-60	3,85	96,16
61-90	3,82	95,44
91-120	3,82	95,49
121-164	3,83	95,78
Average	3,84	95,90

The results of the questionnaire in Table 4 above, show that the students’ response to the use of EduHYPE-IPS learning reached 95.9% (164 students) with the category of very practical, this percentage was obtained by converting the average score on a 4-point Likert scale into a percentage using the formula: (total score obtained ÷ maximum possible score) × 100%. Indicating that EduHYPE-IPS is easy to use and in accordance with the needs of students in social studies learning. Meanwhile, the overall student response was also in the practical category of 3.66% (6 students) to very practical 96.34% (158 students), which illustrates that EduHYPE-IPS is interesting, easy to understand, and helpful in the learning process. These findings confirm that the EduHYPE-IPS developed has a high level of practicality so that it is suitable for use in social studies learning activities.

**The effectiveness test**, the effectiveness test of EduHYPE-IPS was carried out through a pretest and posttest of the creative thinking ability of elementary school students. The effectiveness test was carried out in four elementary schools, namely SDN SLW and SDN CMS in Majalengka Regency as well as SDN PJ and SDN TR in Gunung Kidul district, Yogyakarta with 124 students. Normality and homogeneity tests are carried out to determine parametric or non-parametric tests so that valid and reliable data are obtained. The results of the normality and homogeneity test can be seen in Table 5 (a) Test of Normality, (b) Test of Homogeneity of Variance.

The results of the normality test ( Table 5a) showed that all groups had a significance value of < 0.05, so the data was not distributed normally. Meanwhile, the variance homogeneity test ( Table 5b) also produced a significance value of < 0.05, which means that the data are not homogeneous. Thus, the analysis cannot use parametric tests and is more precisely performed with non-parametric tests.

**Table 5a.** Test of normality.

Class		Kolmogorov-Sminov			Shapiro-Wilk		
		Statistic	df	Sig.	Statistic	df	Sig.
Creative Thinking	Pretest_control	.1677	62	.000	.941	62	.005
	Posttest_control	.137	62	.006	.944	62	.007
	Pretest_experiment	.219	62	.000	.877	62	.000
	Posttest_experiment	.189	62	.000	.916	62	.000

**Table 5b.** Test of homogeneity of variance.

Levene statistic			df1	df2	Sig.
Creative Thinking	Based on Mean	7.246	3	244	.000
	Based on Median	5.395	3	244	.001
	Based on Median and with adjusted df	5.395	3	219.480	.001
	Based on trimmed mean	6.564	3	244	.000

The test used is the Mann-Whitney U Test, which is a non-parametric test to compare the differences between two independent groups when the data is not normally distributed. The results of the Mann-Whitney U Test can be seen in Table 6.

**Table 6.** Mann-Whitney U test.

	Creative thinking
Mann-Whitney U	1132.500
Wilcoxon W	3085.500
Z	-3.993
Asymp. Sig. (2-tailed)	.000

Based on the results of the analysis using the Mann-Whitney U test, the value of U = 1132,500, value Z = -3,993, and the significance value of Asymp were obtained. Sig. (2-tailed) = 0.000. Since the significance value is less than 0.05, it can be concluded that there is a significant difference in creative thinking skills between the two classes, namely the experimental class and the control class.

The Normalized Gain (N-Gain) test in this study was used to determine the effectiveness of EduHYPE-IPS on learning by looking at the improvement of students’ creative thinking skills from pretest to posttest in experimental and control classes. The results of the N-Gain test can be seen in Table 7.

**Table 7.** NGain test results.

NGain_percen	Mean	95% confidence interval for mean	Median	Std. Deviation	Minimum -Maximum	Skewness & Kurtosis
Control class	22.46%	19.26 – 25.65	22.50	12.59	0.00 – 50.00	-0.047 and -0.463
Experiment class	51.81%	45.57 – 58.06	53.57	24.58	0.00 – 87.50	-0.481 and -0.594

Based on the results of the descriptive analysis of Table 7, it was obtained that the average *N-Gain* value in the control class was 22.46% with a 95% confidence interval range between 19.26 to 25.65. Meanwhile, the average *N-Gain* value in the experimental class was much higher, which was 51.81% with a 95% confidence interval between 45.57 and 58.06. This difference showed that the increase in creative thinking ability in the experimental class was more than double compared to the control class. The experimental class showed an increase in creative thinking ability more than twice that of the control class. This improvement was due to the differentiated Hypermedia E-Module, which kept students engaged and encouraged diverse problem-solving approaches. These findings imply that integrating differentiated hypermedia can foster creative thinking and support more inclusive, student-centered learning.

The median in the control class was 22.50 and in the experimental class was 53.57, which was relatively in line with the average value, so the data distribution tended to be stable. In terms of spread, the standard deviation of the control class of 12.59 indicates more homogeneous data, while the experimental class has a standard deviation of 24.58 which indicates more varied data. However, the maximum achievement of the experimental class reached 87.50, much higher than the control class which was only 50.00. The skewness value in both classes was close to zero, which means that the distribution of data was relatively symmetrical, although in the experimental class there was a tendency for more students to achieve high achievements. Thus, descriptively it can be concluded that learning in the experimental class using EduHYPE-IPS had a more significant increase in creative thinking than the control class, although with a more diverse variety of outcomes.

## Discussion

The results of the validation of material, media, and language experts showed an average score of 92.45% with a very valid category. This means that the EduHYPE-IPS developed is in accordance with the content standards, language, and appearance of learning media. Expert validation ensures that social studies content is relevant to learning outcomes, clear material presentation, and interactive and accessible media design. These findings are in line with research ( Adawiyah et al., 2020) and ( Lestari et al., 2022) which emphasizes the importance of expert validation in ensuring the feasibility of digital-based e-modules. Thus, from the aspect of validity, EduHYPE-IPS is suitable for use in social studies learning in elementary schools.

The practicality of the product is seen from the responses of teachers and students. The results of the questionnaire showed that the teacher’s response reached 92.9% in the very practical category, while the student response of 95.9% was also in the very practical category. The teacher assessed that this module was easy to use, in accordance with the characteristics of differentiated learning, and helped in the implementation of social studies learning. Meanwhile, students find the modules interesting, easy to understand, and provide a learning experience according to their interests and learning styles. These findings support the results of the study Gheyssens et al. (2022) which shows that differentiation-based digital learning media is able to increase student engagement. Thus, the practicality aspect shows that EduHYPE-IPS can not only be applied, but also received positive acceptance from teachers and students.

The effectiveness of EduHYPE-IPS was tested through a comparison of experimental and control classes. The results of the Mann-Whitney test showed that there was a significant difference in creative thinking skills between the two groups. In addition, the results of the N-Gain test showed an average increase in the experimental class of 51.81%, much higher than the control class which was only 22.46%. These results indicate that the use of differentiated hypermedia e-modules can increase students’ creative thinking skills more than twice compared to conventional learning. This is in line with research Segundo-Marcos et al. (2025) which affirms that EduHYPE-IPS can strengthen students’ creativity through the interactive integration of text, images, videos, and animations.

Overall, EduHYPE-IPS is declared to be very valid, very practical, and effective. This product not only meets the aspect of academic feasibility, but is also proven to be easy to use by teachers and students, and has a real impact on improving creative thinking skills. Therefore, EduHYPE-IPS can be used as an alternative innovative learning media that is in line with the independent curriculum and 21st century competencies.

## Conclusion

Based on the results of research and development, it can be concluded that the differentiated learning-based hypermedia e-module (EduHYPE-IPS) in elementary school social studies subjects has met the valid, practical, and effective criteria. From the validity aspect, the results of the assessment of material, media, and language experts obtained an average score of 92.45% with a very valid category, so that the product is suitable for use in learning. From the practical aspect, the teacher’s response reached 92.9% and the student’s response was 95.9%, both of which are in the very practical category. This shows that EduHYPE-IPS is easy to use, attractive, and in accordance with the needs of teachers and students in learning. In terms of effectiveness, the results of the Mann-Whitney test showed a significant difference in creative thinking skills between the experimental and control classes. In addition, the N-Gain test showed an average increase of 51.81% in the experimental class, much higher than the control class which was only 22.46%, in the medium category. Thus, EduHYPE-IPS has been proven to be valid in terms of content, practical use in the classroom, and effective in improving students’ creative thinking skills. This product can be used as an innovative alternative in social studies learning in elementary schools while supporting the implementation of the Independent Curriculum and strengthening competencies in the 21st century. This research is still limited to the scope of elementary schools and to certain materials, so further studies are needed to develop similar e-modules at different levels of education, other subjects, or with trials on a wider scale. In addition, advanced research can also add artificial intelligence-based features or learning analytics to personalize the student learning experience in a more immersive way.

## Data Availability

Figures: Figshare. Figure Differentiated Hypermedia E-Module for Social Studies. DOI: https://doi.org/10.6084/m9.figshare.30575789.v1 ( Haryanti et al., 2025b). This project contains the following underlying data:
•
Figure 1. Borg & Gall Research Steps; • Trial using *pretest-posttest control group design;*
• Stages of differentiation-based hypermedia e-module planning; • Prototype e-module hypermedia; • Initial Product Results; • Initial Product Revision Results. Data are available under the terms of the Creative Commons Attribution 4.0 International license (CC-BY 4.0). Tables: Figshare. Table Differentiated Hypermedia E-Module for Social Studies. DOI: https://doi.org/10.6084/m9.figshare.30575951.v1 ( Haryanti et al., 2025d). This project contains the following underlying data:
•
Table (1) Recapitulation of Expert Validation Analysis Results •
Table (2) Limited Scale Product Trial Results •
Table (3) Teacher Response Questionnaire •
Table (4) Student Response Questionnaire •
Table 5(a) Test of Normality •
Table 5(b) Test of Homogeneity of Variance •
Table (6) Mann-Whitney U Test •
Table (7) NGain Test Results Data are available under the terms of the Creative Commons Attribution 4.0 International license (CC-BY 4.0). Figshare. Dataset. Differentiated Hypermedia E-Module for Social Studies (EduHYPE-IPS): Enhancing Creative Thinking Skills of Elementary School Students. DOI: https://doi.org/10.6084/m9.figshare.30454823.v1 ( Haryanti et al., 2025a). This project contains the following underlying data:
• Raw data from the Differentiated Hypermedia E-Module for Social Studies (EduHYPE-IPS) research: Enhancing Creative Thinking Skills of Elementary School Students. Data are available under the terms of the Creative Commons Attribution 4.0 International license (CC-BY 4.0). Figshare. Dataset. Questionnaires and Interview Guides. DOI: https://doi.org/10.6084/m9.figshare.30347200.v2 ( Haryanti et al., 2025c). This project contains the following underlying data:
• To collect comprehensive data regarding the implementation of differentiated instruction and the potential of hypermedia-based learning in Social Studies, two instruments were developed: the Teacher’s Questionnaire Sheet and the Interview Sheet. Data are available under the terms of the Creative Commons Attribution 4.0 International license (CC-BY 4.0) **.**
