# Supplementary material for: Differentiated Hypermedia E-Module for Social Studies (EduHYPE-IPS): Enhancing Creative Thinking Skills of Elementary School Students
Source: F1000Res. 2025 Dec 8;14:1368. [Version 1] doi: 10.12688/f1000research.171479.1 (PMC12796794; doi:10.12688/f1000research.171479.1)
Supplement: Peer review history

# Reviewer report: Reviewer response for version 1

**DOI:** 10.5256/f1000research.189090.r440211

**Published:** 2026-01-12

**Authors:** Dewi Hikmah Marisda

**Affiliations:** 1 Universitas Muhammadiyah Makassar, Makassar, South Sulawesi, Indonesia

This article presents a development study of a differentiated learning–based hypermedia e-module designed to enhance elementary school students’ creative thinking skills. The manuscript is clearly written, well-structured, and supported by up-to-date and relevant literature. The Research and Development design employing the Borg & Gall model is implemented systematically, starting from needs analysis through to product effectiveness testing. Expert validation procedures, practicality testing involving teachers and students, and effectiveness testing using a pretest–posttest control group design are described in sufficient detail to allow replication.

 The statistical analysis is appropriately conducted with due consideration of data characteristics, including the use of non-parametric Mann–Whitney U tests and N-Gain calculations. The interpretation of the results is consistent and demonstrates that the EduHYPE-IPS e-module is significantly more effective than conventional instruction in improving students’ creative thinking skills. All supporting data are openly available through public repositories, ensuring transparency and reproducibility. The conclusions are well aligned with the findings, and the study’s limitations are acknowledged proportionally. Overall, this article meets scientific standards and is suitable for indexing.

 I have selected the decision **“Approved with Reservations,”** indicating that the manuscript shows potential but requires further revisions before it can be considered scientifically valid. The authors are advised to address the reviewer comments, particularly regarding clarity, methodological consistency, and strengthening the analysis.

Is the work clearly and accurately presented and does it cite the current literature?

Yes

If applicable, is the statistical analysis and its interpretation appropriate?

Yes

Are all the source data underlying the results available to ensure full reproducibility?

Yes

Is the study design appropriate and is the work technically sound?

Yes

Are the conclusions drawn adequately supported by the results?

Yes

Are sufficient details of methods and analysis provided to allow replication by others?

Yes

Reviewer Expertise:

Physics Education, Science Education, Digital Learning Media, Instructional Design, Higher Order Thinking Skills, Differentiated Learning

I confirm that I have read this submission and believe that I have an appropriate level of expertise to confirm that it is of an acceptable scientific standard, however I have significant reservations, as outlined above.

# Reviewer report: Reviewer response for version 1

**DOI:** 10.5256/f1000research.189090.r440216

**Published:** 2026-01-12

**Authors:** Deny Hadi Siswanto

**Affiliations:** 1 Muhammadiyah Mlati Senior High School, Mlati, Sleman, Indonesia

**Additional suggestions on the background:**

**1. Integration of Differentiation:** The hypermedia e-module should adapt content, learning processes, and products to students’ individual characteristics to enhance relevance, motivation, and creativity.

 2. **Development of Creative Thinking:** The module should stimulate interactive and problem-based learning activities that foster creative thinking as a core 21st-century competency.

 3. **Comprehensive Module Evaluation:** The validity, practicality, and effectiveness of the module should be strengthened through long-term evaluation and contextual analysis to ensure adaptability across diverse learning environments.

Is the work clearly and accurately presented and does it cite the current literature?

Yes

If applicable, is the statistical analysis and its interpretation appropriate?

Yes

Are all the source data underlying the results available to ensure full reproducibility?

Yes

Is the study design appropriate and is the work technically sound?

Yes

Are the conclusions drawn adequately supported by the results?

Yes

Are sufficient details of methods and analysis provided to allow replication by others?

Yes

Reviewer Expertise:

-

I confirm that I have read this submission and believe that I have an appropriate level of expertise to confirm that it is of an acceptable scientific standard.

# Reviewer report: Reviewer response for version 1

**DOI:** 10.5256/f1000research.189090.r440212

**Published:** 2026-01-02

**Authors:** eny winaryati

**Affiliations:** 1 Universitas Muhammadiyah Semarang, Semarang, Central Java, Indonesia

The manuscript is clearly presented and well structured, with appropriate use of current and relevant literature. The study design is suitable for addressing the research objectives and is technically sound, with adequate controls included where necessary. The methods and analytical procedures are described in sufficient detail to allow replication, and relevant reporting guidelines have been followed. The statistical analysis is appropriate and correctly interpreted. All underlying source data are available and adequately documented, ensuring reproducibility. The conclusions are well supported by the results, and the limitations are appropriately acknowledged. Overall, the study is scientifically sound and suitable for indexing.

Is the work clearly and accurately presented and does it cite the current literature?

Yes

If applicable, is the statistical analysis and its interpretation appropriate?

Yes

Are all the source data underlying the results available to ensure full reproducibility?

Yes

Is the study design appropriate and is the work technically sound?

Yes

Are the conclusions drawn adequately supported by the results?

Yes

Are sufficient details of methods and analysis provided to allow replication by others?

Yes

Reviewer Expertise:

NA

I confirm that I have read this submission and believe that I have an appropriate level of expertise to confirm that it is of an acceptable scientific standard.
